# Uveal Melanoma Cells Elicit Retinal Pericyte Phenotypical and Biochemical Changes in an in Vitro Model of Coculture

**DOI:** 10.3390/ijms21155557

**Published:** 2020-08-03

**Authors:** Carmelina Daniela Anfuso, Anna Longo, Alfio Distefano, Angela Maria Amorini, Mario Salmeri, Guido Zanghì, Cesarina Giallongo, Giovanni Giurdanella, Gabriella Lupo

**Affiliations:** 1 Department of Biomedical and Biotechnological Sciences, School of Medicine, Section of Medical Biochemistry, University of Catania, 95123 Catania, Italy; daniela.anfuso@unict.it (C.D.A.); longo.anna@hotmail.it (A.L.); distalfio@gmail.com (A.D.); amorini@unict.it (A.M.A.); gabriella.lupo@unict.it (G.L.); 2 Department of Biomedical and Biotechnological Sciences, School of Medicine, Section of Microbiology, University of Catania, 95123 Catania, Italy; msalmeri@unict.it; 3 Department of Surgery, Policlinico Vittorio Emanuele University Hospital - General Surgery and Oncology Unit, University of Catania, 95123 Catania, Italy; gzanghi@unict.it; 4 Department of Medical and Surgical Science and Advanced Technologies “G.F. Ingrassia”, University of Catania, 95123 Catania, Italy; cesarinagiallongo@yahoo.it

**Keywords:** coculture, pericytes, PDGF-B, uveal melanoma, STAT3

## Abstract

Vascular pericytes are an important cellular component in the tumor microenvironment, however, their role in supporting cancer invasion is poorly understood. We hypothesized that PDGF-BB could be involved in the transition of human retinal pericytes (HRPC) in cancer-activated fibroblasts (CAF), induced by the 92.1 uveal melanoma (UM) cell line. In our model system, HRPC were conditioned by co-culturing with 92.1UM for 6 days (cHRPC), in the presence or absence of imatinib, to block PDGF receptor-β (PDGFRβ). The effects of the treatments were tested by wound healing assay, proliferation assay, RT-PCR, high-content screening, Western blot analysis, and invasion assay. Results showed profound changes in cHRPC shape, with increased proliferation and motility, reduction of NG2 and increase of TGF-β1, α-SMA, vimentin, and FSP-1 protein levels, modulation of PDGF isoform mRNA levels, phospho-PDGFRβ, and PDGFRβ, as well as phospho-STAT3 increases. A reduction of IL-1β and IFNγ and an increase in TNFα, IL10, and TGF-β1, CXCL11, CCL18, and VEGF mRNA in cHRPC were found. Imatinib was effective in preventing all the 92.1UM-induced changes. Moreover, cHRPC elicited a significant increase of 92.1UM cell invasion and active MMP9 protein levels. Our data suggest that retinal microvascular pericytes could promote 92.1UM growth through the acquisition of the CAF phenotype.

## 1. Introduction

### 1.1. Pericytes in the Tumor Microenvironment

The tumor microenvironment (TME) is constituted by both the extracellular matrix and multiple infiltrating cell components including smooth muscle cells, pericytes, endothelial cells, mesenchymal stem cells, a variety of immune cells and cancer-associated fibroblasts (CAFs) [1,2,3]. Dynamic and bidirectional crosstalk between TME and cancer cells plays a key role in the processes of tumor growth and invasiveness, destabilizing tissue homeostasis by modifying pH, promoting angiogenesis, and secreting several molecules including metabolites, cytokines, and chemokines [4,5,6,7]. Pericytes are mainly described as “mural” cells, specialized in vascular homeostasis, and they participate in angiogenesis by regulating the remodeling and stabilization of the blood–brain and blood–retina barriers [8]. Cell plasticity of pericytes is also described since they can differentiate into different cell types including phagocytes, chondrocytes, adipocytes, myocytes, and osteoblasts and can contribute to naturally occurring regenerative processes [8,9]. Vascular pericytes are considered an important source of CAFs, the most critical and abundant components of the tumor mesenchyme [5,10,11].

### 1.2. Pericyte Markers in Physiological and Pathological Conditions

Pericytes express several antigenic markers that are useful to allow their characterization that commonly includes the neural/glial proteoglycan antigen 2 (NG2), the platelet-derived growth factor receptor-β (PDGFRβ), CD146, and alpha-smooth muscle actin (α-SMA) [12,13]. Recently, the interest in studying pericyte involvement in both healthy and pathological processes has grown rapidly. Despite the biological potential of pericytes, their dysfunction can contribute to the pathogenesis of atherosclerosis, fibrosis, vascular calcification, diabetic retinopathy, and tumor progression [14,15,16,17]. In this regard, following the interaction with glioblastomas, pericytes acquire an anti-inflammatory and immunosuppressive phenotype with high levels of anti-inflammatory cytokines IL-10 and TGF-β1 and a low level of the pro-inflammatory cytokine TNFα, thus helping immune evasion and proliferation of the tumor [18]. In addition, CAFs markedly promote tumor metastases, as they secrete many growth factors and matrix degrading enzymes that result in a remodeling of the ECM, intravasation of metastatic cancer cells in blood or lymphatic vessels, colonization, and establishment [6,19]. Activated CAFs express molecular markers such as vimentin, α-smooth muscle actin (α-SMA), fibroblast activation protein α (FAPα), and fibroblast specific protein-1 (FSP-1) [20,21].

### 1.3. PDGF-BB/PDGFRβ Signaling in Pericyte-CAF Transition

Pericyte-CAF transition is mediated by PDGF-BB/PDGFRβ signaling through the mechanism of pericyte–fibroblast transition (PFT) [10]. The PDGF family consists of four different polypeptide chains that assemble to form homodimers (PDGF-AA, -BB, -CC, and -DD) and a heterodimer PDGF-AB [22]. The PDGR isoforms exert their cellular effects by interacting with tyrosine kinases PDGF receptors (PDGFR) α and β. Ligand-binding to PDGFR induces receptor dimerization, leading to the formation of PDGFR-αα, -αβ, and -ββ dimers and the autophosphorylation on tyrosine residues of the intracellular kinase domain [23,24]. Cellular effects of PDGFR activation are triggered by the involvement of several pathways including tyrosine kinases of the Src family, phosphatidylinositol-3′-kinase (PI3K), phospholipase C-γ1 (PLC-γ1), the Grb2/Sos1 –Ras axis, the ERK MAP kinase pathway, the tyrosine phosphatase SHP-2, and the signal transducer and activator of the transcription STAT family [25,26,27]. PDGF and/or PDGF receptors are overexpressed or mutated in different solid tumors and participate in tumor progression through autocrine stimulation of tumor cell growth and paracrine stimulation on stromal cells to generate a favorable microenvironment for tumor survival [28]. A well-known molecular mechanism of CAF production is associated with STAT3 activation through the autocrine secretion of the IL-6 pro-inflammatory cytokine member family LIF upon TGF-β1 stimulation [29,30]. Moreover, STAT3 activation was described to be strongly induced by PDGF-mediated phosphorylation of conserved serine residue at position 727 [31,32,33]. High expression of PDGFRβ and high content of CAFs in the tumor stroma are related to an unfavorable prognosis in breast and prostate cancer [34,35], colorectal cancer [15,36,37], and pancreatic carcinoma [38]. Tumor-derived PDGF-BB was described to promote pericyte transition into CAF stimulating tumor growth and metastasis “in vivo,” assuming that pericyte-fibroblast transition also occurs in human tumors. [10]. Moreover, it could potentially attract pericytes from vessels through a chemoattractant gradient mechanism so as to drive pericyte recruitment towards the tumor [10,39].

### 1.4. Involvement of Pericytes in Uveal Melanoma Progression

Uveal melanoma (UM) is the most common primary intraocular tumor with an incidence of approximately 2–8 per million per year in Caucasians [40,41]. Metastatic UM is associated with hepatic lesions in up to 90% of cases with a mortality rate of 92% within 2 years and an overall survival spanning from 3 to 16 months [42,43,44]. UM has been described to affect the chemotactic response and maturation of monocytes in vitro [45]. UM cell composition includes malignant tumor cells and inflammatory cells, such as macrophages, lymphocytes, and fibroblasts [46]. New synergistic and multitarget therapeutic strategies for uveal melanoma treatment can derive from a better understanding of tumor biology [47] and the functional contribution of the tumor microenvironment. Most UM are located posterior to the equator of the eye and adjacent to the retina leading to serous foveal detachment, photoreceptor degeneration, subretinal choroidal neovascularization, or edema [46]. Inhibition of pericytes in NG2 knockout mice decreased neovascularization and tumor volume, indicating an important role of pericytes in the progression of uveal melanoma [48]. Considering the high density of pericyte covering the retinal capillaries proximal to the tumor [49], as well as their pivotal role in maintaining blood-retinal barrier (BRB) integrity [50], we hypothesized retinal pericytes as a source of CAFs in UM. Here, we tested the capability of HRPC to originate CAF following interaction with 92.1 (UM), using an in vitro model of coculture that allowed the reciprocal conditioning through diffusible factors produced by both cell types. The role of the PDGF-BB/PDGFRβ axis blockade in the differentiation of HRPC into CAF was tested by using the specific inhibitor imatinib in a coculture medium.

## 2. Results

### 2.1. PDGF-BB is Involved in the Modulation of Pericyte Marker Protein Levels in HRPC Conditioned by 92.1UM

**Figure 1:**
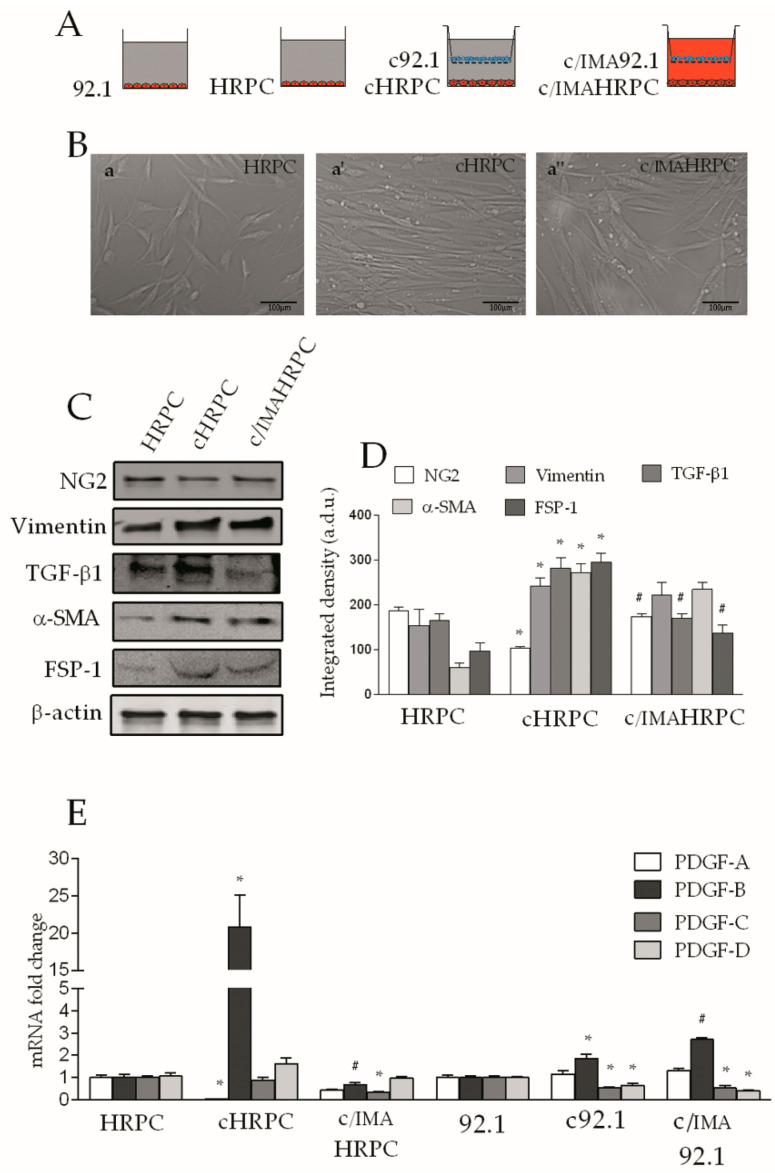
Imatinib prevents marker expression changes in human retinal pericytes (HRPC) induced by the interaction with 92.1 uveal melanoma (UM) cells. Human retinal pericytes were grown alone (control HRPC) or were conditioned in the presence of 92.1UM (cHRPC) with or without 5 μM of imatinib (c/IMAHRPC) for 6 days (**A**,**B**): representative images of HRPC (**a**), cHRPC (**a’**), and c/IMAHRPC (**a’’**) after 6 days of conditioning. (**C**): Western blot analysis of NG2, vimentin, TGF-β1, α-SMA, and FSP-1 proteins in control pericytes (HRPC), cHRPC, and c/IMAHRPC. β-actin detection indicates the same loading of 30 μg of protein in each lane. (**D**): immunoblot quantification of through densitometric analysis of each band (in arbitrary densitometry units, a.d.u.), carried out with the Image J program. (**E**): quantification of PDGF-A, PDGF-B, PDGF-C, and PDGF-D mRNA expression levels by qRT-PCR in both HRPC and 92.1UM grown alone and in coculture with or without 5 μM of imatinib. Bars represents the means ± SEM from three independent experiments. * *p* < 0.05 vs. control (HRPC or 92.1UM); # *p* <0.05 vs. coculture without imatinib. One-way ANOVA, followed by Tukey’s test.

We first aimed to determine if (i) pericyte-CAF transition would occur in response to the interaction with 92.1UM uveal melanoma in an in vitro model of coculture. As described in Section 2.2, HRPC were grown for 6 days alone (HRPC) or conditioned in the presence of 92.1UM (cHRPC). Moreover, (ii) in order to test the role of PDGF-BB in 92.1UM-induced pericyte-CAF transition, pericytes were also cocultured in the presence of 5 μM imatinib (c/IMAHRPC), a tyrosine kinase inhibitor (TKI) used as an inhibitor of PDGFR signaling (Figure 1A).

HRPC were previously validated by the positivity for typical pericyte markers, such as α-SMA, PDGFRβ, and NG2, and for the absence of the endothelial marker, vWF [51]. After conditioning, analysis of morphology based on phase-contrast microscopy showed a profound change of cellular shape in cHRPC as indicated by an elongated cell body and a reduction of the cell extensions typical of pericytes in comparison to control HRPC (Figure 1B, a’, and a). In c/IMAHRPC, a more similar cell shape to typical pericyte morphology was observed, including a reduction of cell body elongation, more irregular shape, longer processes, and large, flat cell bodies compared to cHRPC (Figure 1B, a’’). Western blot analysis of the typical pericyte markers (α-SMA and NG2) showed a significant reduction of the NG2 protein level of about 40% in cHRPC; this effect was eliminated by 5 μM of imatinib in coculture medium that restored the NG2 protein to the control levels (Figure 1C,D). Coculture also increased α-SMA protein levels by about fivefold (*p* < 0.01) in comparison to control HRPC. Overall, 5 μM of imatinib in coculture medium did not significantly affect upregulation of α-SMA protein levels. CAF marker vimentin, TGF-β1, and FSP-1 protein levels were significantly increased in HRPC cocultured with 92.1UM for 6 days in comparison to HRPC (about 1.5, 1.8, and 3.2-fold change, respectively, *p* < 0.05). Specifically, 5 μM of imatinib in coculture medium did not significantly affect upregulation of vimentin protein levels, whereas it strongly reduced both TGF-β1 and FSP-1 protein levels in comparison to cHRPC (Figure 1C,D). These data indicate a clear change of the HRPC phenotype towards a cancer-activated fibroblast-like phenotype. As PDGF-BB-PDGFR signaling significantly contributes to the increase of CAFs in human tumors [10], we analyzed the mRNA of PDGF-A, PDGF-B, PDGF-C, and PDGF-D mRNA isoforms by real-time RT-PCR in both HRPC and 92.1UM cocultured for 6 days, in order to discriminate the reciprocal contribution in PDGF isoform production of both cell lines. cHRPC showed both a reduction of PDGF-A mRNA levels of about 90% (*p* < 0.05) and, interestingly, a very strong induction of mRNA levels of PDGF-B (*p* < 0.5) in comparison to control HRPC (Figure 1D). The presence of imatinib (5 μM) in the coculture medium eliminated both the reduction of PDGF-A and the increase of PDGF-B mRNA levels as well as reduced PDGF-C mRNA levels in comparison to c/IMAHRPC. Real-time RT-PCR analysis showed a significant increase in PDGF-B mRNA levels of about 0.9- and 1.7-fold in c92.1 and c/IMA92.1, respectively (*p* < 0.05) compared to control cells. Moreover, a significant reduction in PDGF-C and –D mRNA levels of about 50% in both c92.1 and c/IMA92.1 was observed in comparison to controls (92.1). No significant differences were detected in PDGF-A mRNA levels (Figure 1D). These data are consistent with a PDGF-BB-mediated phenotype transition of HRPC towards a cancer-activated fibroblast-like phenotype in response to 92.1UM cell interaction.

### 2.2. Imatinib Blocked the Enhanced HRPC Migration and Proliferation Induced by 92.1UM

**Figure 2:**
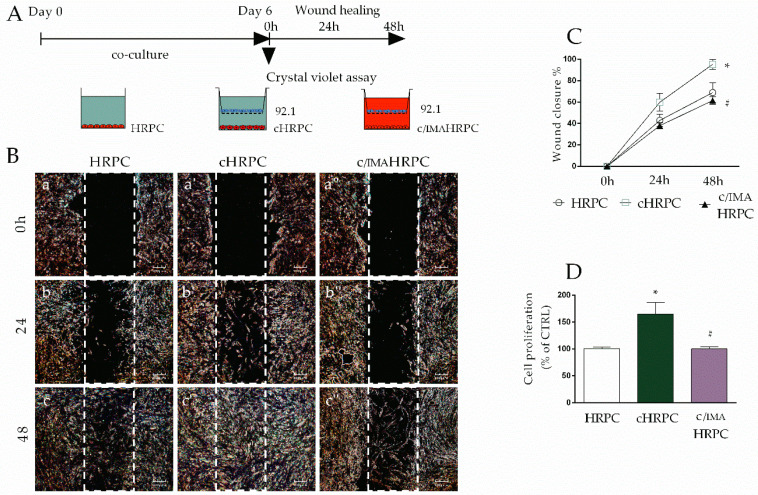
Imatinib reduces the increased cell migration and proliferation in human retinal pericytes (HRPC) induced by 92.1UM. Cell migration and proliferation were evaluated in HRPC cultured alone (control, HRPC) or in the presence of 92.1 without (cHRPC) or with 5 μM of imatinib (c/IMAHRPC) for 6 days. (**A**): schematic representation of performed experimental model. (**B**): wound healing assays with representative images of control HRPC, cHRPC, and c/IMAHRPC immediately after the scratch at 0 h (**a**, **a’** and **a’’**, respectively), after 24 h (**b**, **b’,** and **b’’**, respectively), and after 48 h of incubation (**c**, **c’,** and **c’’**, respectively). Percentage of wound closure was quantified by Image J software (**C**). Crystal violet assays were carried out in coculture after 6 days of conditioning. (**D**). All data represent mean ± SEM obtained from at least three independent experiments * *p* < 0.05 vs. control (HRPC); # *p* < 0.05 vs. coculture conditions without imatinib. One-way ANOVA, followed by Tukey’s test.

As tumor activation of fibroblasts triggers both high migration and proliferation rates in patients and in animal models [52,53], we next aimed to evaluate the migratory ability and proliferation rate in pericytes after 6 days of interaction with 92.1UM cells. For this reason, a wound-healing assay was performed at the end of the conditioning period as described in the Materials and Methods section. As reported in Figure 2, migratory ability was greatly enhanced in cHRPC, enough to close the wound already at 48 h, whereas control HRPC resulted in a wound closure of about 50% (Figure 2A,B).

This effect was completely reverted by the presence of 5 μM imatinib in the coculture medium (c/IMAHRPC) that brought migratory capacity back to the level of control pericytes. Next, we analyzed cell proliferation after 6 days of conditioning by 92.1UM and analogous data were observed. Indeed, cHRPC showed a significant increase in cell proliferation of about 65% (*p* < 0.05) in comparison to HRPC, whereas 5 μM of imatinib in coculture media restored the proliferation rate to control levels (Figure 2C). These data indicate that PDGF signaling could drive the increase of HRPC migration and proliferation induced by 92.1 uveal melanoma.

### 2.3. Long-Lasting Interaction with 92.1UM Induce Pericytes Switch a Pro- to Anti-Inflammatory Phenotype, an Effect Reverted by Imatinib

**Figure 3:**
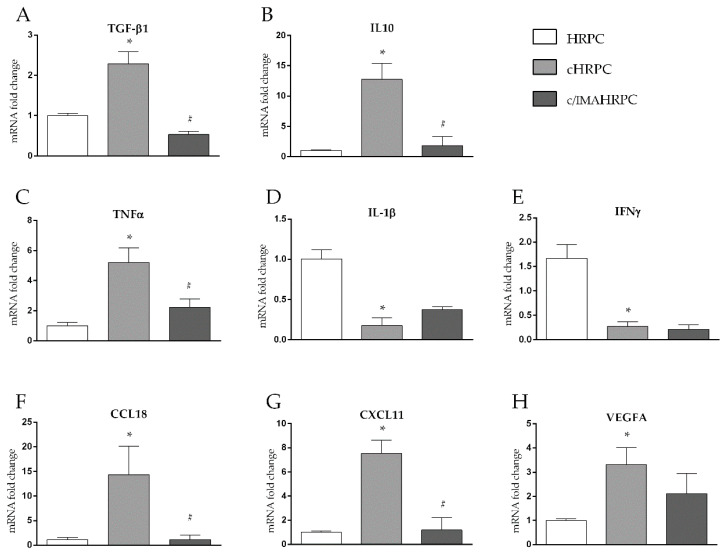
HRPC interacting with 92.1UM show an anti-inflammatory marker profile, an effect reduced by imatinib. Quantitative analysis of TGF-β1 (**A**), IL10 (**B**), TNFα (**C**), IL-1β (**D**), IFNγ (**E**), CCL18 (**F**), CXCL11 (**G**), and VEGF (**H**) mRNA levels in pericytes cultured alone (HRPC) or in the presence of 92.1UM without (cHRPC) or with 5 μM of imatinib (c/IMAHRPC) for 6 days. Results are relative to those of basal levels in control HRPC cultured for 6 days alone and normalized to the housekeeping reference gene ribosomal 18S RNA expression. All data represent mean ± SEM obtained from three independent experiments. * *p* < 0.05 vs. control (HRPC); # *p* < 0.05 vs. coculture conditions without imatinib. One-way ANOVA, followed by Tukey’s test.

To verify if activated HRPC may acquire a pro- or anti-inflammatory functions in response to the interaction with uveal melanoma, inflammatory cytokine mRNA expression levels in HRPC cultured alone or cocultured in the presence of 92.1UM without (cHRPC) or with 5 μM of imatinib (c/IMAHRPC) for 6 days were evaluated. Quantitative RT-PCR analyses showed a significant increase in TGF-β1 mRNA levels (about 1.5-fold change, *p* < 0.05) confirming the increased protein levels of TGF-β1, previously shown in Figure 1B,C and Figure 3A.

Moreover, a strong increase of IL10 mRNA levels (about 11.5-fold change, *p* < 0.05) in cHRPC compared to control HRPC (Figure 3B) was observed. The presence of 5 μM of imatinib in coculture completely prevented the increase of both the anti-inflammatory cytokines induced by 92.1UM as evidenced by mRNA levels of TGF- β1 and IL10 in c/IMAHRPC similar to control HRPC. Interestingly, 6 days of coculture with 92.1UM triggered a significant increase of pro-inflammatory cytokine TNF-α mRNA of about 3.5-fold (*p* < 0.05) and a reduction of IL-1β and IFN- γ mRNA levels of about 75% and 85%, respectively (*p* < 0.05, Figure 3C–E). HRPC cocultured with 92.1UM in the presence of imatinib (5 μM) showed restored levels of mRNA only for TNF-α, whereas they remained unchanged for IL-1β and IFN-γ in comparison to cHRPC. Overall, these data indicate a modulation of inflammation genes in favor of an immunomodulatory phenotype. Next, we tested the involvement of chemokines CXC11 and CCL18 as well as VEGFA as they were found involved in uveal melanoma recruitment of tumor-associated microphages and angiogenesis [54,55]. Interestingly, quantitative RT-PCR analysis revealed a strong increase of CXC11, CCL18, and VEGFA mRNA levels in HRPC after 6 days of coculture with 92.1UM in comparison to control HRPC (of about 13, 7, and 3-fold changes, respectively, *p* < 0.05) (Figure 3F–H). Moreover, this induction was completely reduced by the presence of 5 μM imatinib in the coculture medium for CXCL11 and CCL18 mRNA levels and only partially for VEGFA levels (Figure 3F–H). These data suggest that PDGF could mediate pericytes acquisition of both the immune-modulatory function and tumor progression capacity in response to uveal melanoma interaction.

### 2.4. Imatinib Prevented 92.1UM-Mediated Nuclear Translocation of Phospho-STAT3 and PDGFRβ Activation in HRPC

**Figure 4:**
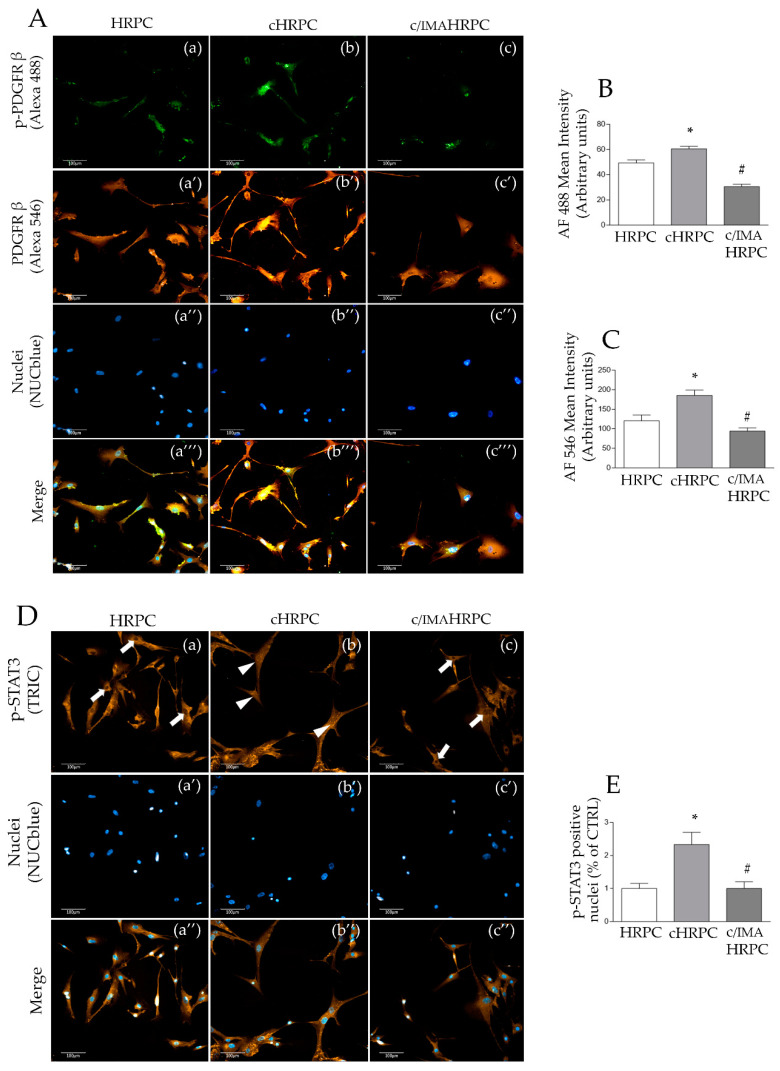
Immunocytochemical staining associated with high-content screening was used to analyze phospho-PDGFRβ (p-PDGFRβ), total PDGFRβ, and phospho-STAT3 (p-STAT3), in HRPC cultured alone (control, HRPC) or in the presence of 92.1UM (cHRPC) without or with 5 μM of imatinib (c/IMAHRPC) for 6 days. (**A**): representative images of double immunocytochemical stained HRPC, cHRPC, and c/IMAHRPC for p-PDGFRβ (in green; **a**, **b,** and **c**, respectively) and PDGFRβ (in red; **a’’**, **b’’,** and **c’’**, respectively). Moreover, relative NucBlue-stained nuclei (**a’’**, **b’,** and **c’**, respectively) and merged channels (**a’’’**, **b’’’,** and **c’’’**, respectively) are shown. High-content screening analysis was used to quantify green fluorescence (Alexa Fluor 488) relative to p-PDGFRβ staining (**B**) and red fluorescence (Alexa Fluor 546) relative to total PDGFRβ staining (**C**,**D**): representative images relative to immunostaining for p-STAT3 (red) in HRPC, cHRPC, and c/IMAHRPC (**a**, **b,** and **c**, respectively), the NucBlue-stained nuclei (**a’**, **b’,** and **c’**, respectively), and merged channels (**a’’**, **b’’,** and **c’’**, respectively). White arrowheads indicate p-STAT3-negative nuclei, whereas white arrows indicate p-STAT3-positive nuclei. (**E**): quantification of p-STAT3-positive nuclei in HRPC, cHRPC, and c/IMAHRPC carried out by high-content screening analysis (Section 4.7). All images were acquired with the Operetta High-Content Imaging System using a 20× magnification; scale bar = 100 μm. Data are expressed as media ± SEM from at least six fields/well randomly selected and each reporting more than 15 cells/field. All data represent mean ± SEM obtained from at least three independent experiments. * *p* < 0.05 vs. control (HRPC); # *p* < 0.05 vs. coculture conditions without imatinib. One-way ANOVA, followed by Tukey’s test.

As 92.1UM promotes retinal pericyte-CAF transition of HRPC mediated by PDGF-B, leading to a putative immune-modulatory function as well as tumor progression capacity, phospho- and total PDGFRβ protein levels were analyzed in order to validate the involvement of PDGR-BB activity in our model. High-content screening analysis showed a slight but significant increase of immunoreactivity for phospho-PDGFRβ in HRPC cocultured with 92.1UM compared to control HRPC as indicated by the increased green fluorescence (Figure 4A).

Moreover, the presence of imatinib in the coculture medium (c/IMAHRPC) triggered a reduction of green fluorescence staining in comparison to cHRPC, lower than control (HRPC) levels, indicating the reduction of phospho-PDGFRβ (Figure 4A(a–c),B). Similar data were obtained by the immunostaining for total PDGRβ. Increased levels of PDGFRβ were observed in cHRPC of about 50% (*p* < 0.05) compared to control HRPC as indicated by the levels of red immunostaining; these effects were blocked by imatinib (Figure 4A(a’–c’),B). These data indicate the involvement of PDGFRβ in retinal pericyte-CAF transition induced by 92.1UM. Moreover, we analyzed the nuclear translocation of the active STAT3 transcription factor, shown to be involved in the activation processes of cancer-associated fibroblasts [31]. Immunostaining for the phospho-STAT3 (Y702) in cHRPC showed an increase of positive nuclei for the active isoform STAT3 (about 5-fold, *p* < 0.05) in comparison to control HRPC. In c/IMAHRPC samples, the number of positive nuclei for phospho-STAT3 was similar to the positive nuclei observed in HRPC. These data suggest that the phenotype reprogramming observed in HRPC-CAF transition could be mediated by a STAT3 nuclear translocation induced by PDGFRβ activation.

### 2.5. Imatinib Prevented 92.1UM-Mediated Nuclear Translocation of Phospho-STAT3 and PDGFRβ Activation in HRPC

**Figure 5:**
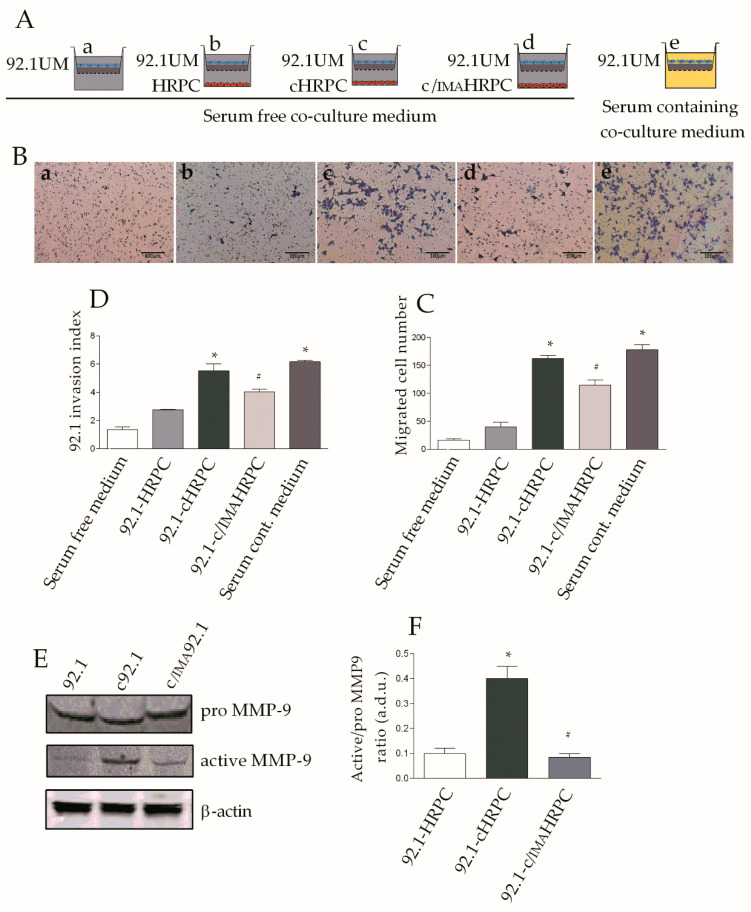
Role of imatinib in the prevention of 92.1UM increasing invasiveness induced by conditioned HRPC. Invasion assay was used to detect the effect of conditioned pericytes on 92.1UM invasiveness. Human retinal pericytes were grown on the lower well alone (HRPC) or cocultured with 92.1UM (seeded into the transwells) without (cHRPC) or with 5 μM of imatinib (c/IMAHRPC) for 6 days. Then, invasion assays were carried out by replacing the transwells with Boyden chambers with fresh 92.1UM and incubating for 24 h. (**A**): diagram representing in vitro model. (**B**): representative images of basolateral Boyden chamber obtained using an inverted optical microscope showing migrated 92.1UM through the membranes in response to serum-free coculture medium as negative control (**a**), control HRPC (**b**), cHRPC (**c**), and c/IMAHRPC (**d**) and in the presence of serum-containing coculture medium as positive control (**e**). (**C**): number of migrated cells counted in randomly selected fields. (**D**): trypan blue quantification of stained 92.1UM after migration (migration index) through optical density evaluation. (**E**): Western blot analysis of pro- and active MMP9 in 92.1UM after interaction with control HRPC (92.1), cHRPC (c92.1), and c/IMAHRPC (c/IMA92.1), respectively. β-actin detection indicates the same loading in each lane of protein (30 μg) per lane. (**F**): ratio of immunoblot quantification of active vs. pro-MMP9 protein; densitometry analysis of each band (in arbitrary densitometry units, a.d.u.), was carried out with the Image J program. All data are expressed as means ± SEM from three independent experiments. * *p* < 0.05 vs. control (HRPC); # *p* < 0.05 vs. coculture conditions without imatinib. One-way ANOVA, followed by Tukey’s test.

To assess HRPC involvement in 92.1UM invasiveness, 92.1UM, seeded on Matrigel-coated filters in a Boyden chambers, were cocultured with HRPC, cHRPC, or c/IMAHRPC (see Materials and Methods section for details) and tumor invasion was evaluated after 24 h (Figure 5A). Boyden chambers were applied to a serum-free coculture medium as negative control, to control HRPC (not conditioned), considered as control samples, whereas 10% FBS was used as chemoattractives in serum-contained coculture medium positive controls. As shown in Figure 5B,C,D, the invasive rate resulted increased in 92.1UM stimulated with cHRPC, as indicated by the significantly increased number of migrated tumor cells (about 3.5-fold, *p* < 0.05) in comparison to 92.1UM stimulated by control (not conditioned) HRPC.

Conditioned HRPC in the presence of 5 μM of imatinib did not trigger the invasion in 92.1UM as indicated by a similar number of migrated 92.1UM in comparison to 92.1UM stimulated by control HRPC. These data were confirmed by the evaluation of the invasion index considered as optical density measurements of trypan blue staining dissolved by migrated tumor cells. In order to better support previous findings, we tested the hypothesis that increased invasiveness of 92.1UM stimulated by conditioned HRPC could be mediated by MMP9 induction. In a parallel set of experiments, 92.1UM were recovered from transwells and total whole lysate was subjected to Western blot analysis to evaluate the active protein levels of MMP9 that mediate Matrigel degradation and invasiveness. As reported in Figure 5E, increased protein levels of active MMP9 were found in 92.1UM stimulated for 24 h by cHRPC in comparison to 92.1UM incubated in the presence of not-conditioned HRPC. Interestingly, this effect was reduced in 92.1UM stimulated by HRPC conditioned in presence of 5 μM imatinib. Overall, these data suggest that cancer activated fibroblast-like phenotypes reached by HRPC after 6 days of exposition to 92.1UM could, in turn, stimulate tumor invasiveness, indicating a putative pathological role of activated HRPC in uveal melanoma progression.

## 3. Discussion

### 3.1. 92.1UM Induce Upregulation of Specific CAF Markers in HRPC

**Figure 6:**
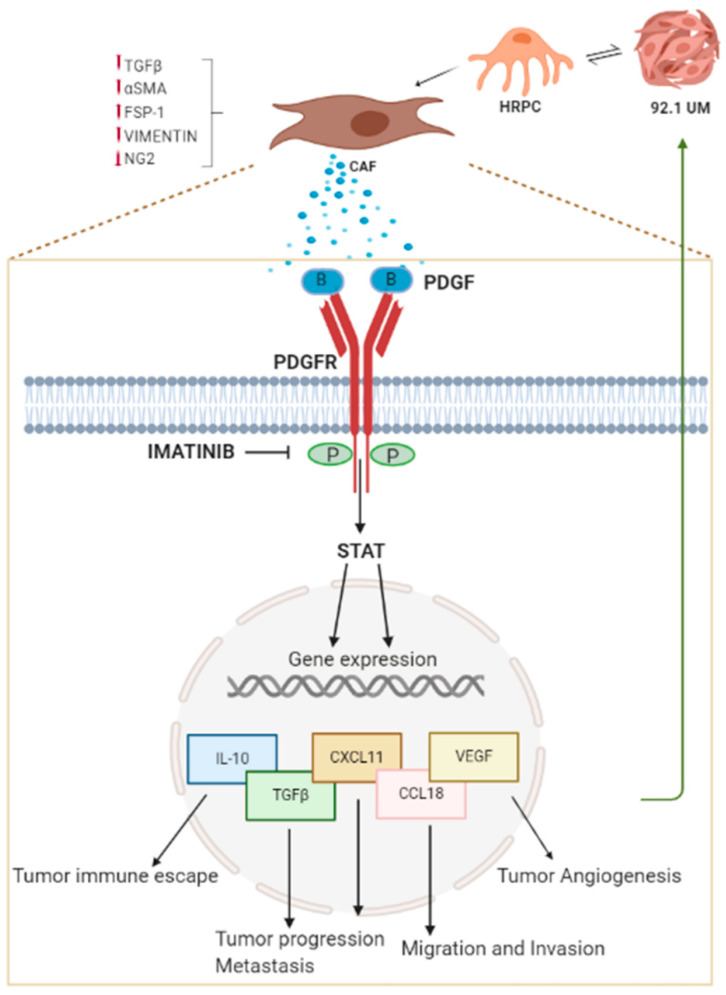
Putative signaling pathway involved in HRPC–CAF transition mediated by PDGF-BB increase. In the proposed model, PDGF-BB is involved in HPRC-CAF transition induced by 92.1UM, confirmed by the significantly increased of CAF markers. PDGF-BB released by activated HRPC binds in an autocrine manner to the receptor PDGFRβ, allowing its activation by dimerization and autophosphorylation. In our model, we verified the involvement of the JAK/STAT pathway as indicated by the nuclear translocation of phospho-STAT3 following activation by 92.1UM. STAT3 transcription factor is known to increase the transcription of specific genes such as IL-10, TGF-β1, CCL10, CXCL11, and VEGF as observed in HRPC cocultured with 92.1UM. Indeed, activated HRPC could support different activities of 92.1UM (proliferation, migration, invasion, angiogenesis, and immune escape) by producing different cytokines and chemokines. Imatinib inhibits PDGFRβ activation and, consequently, CAF transition and tumor invasiveness. Abbreviations: 92.1UM, human uveal melanoma; HRPC, primary human retinal pericytes; CAF, cancer-associated fibroblasts; TGF-β1, transforming growth factor beta; α-SMA, alpha-smooth muscle actin; FSP1, fibroblast-specific protein 1; NG2, neural/glial antigen 2; PDGFR, platelet-derived growth factor receptor; PDGF, platelet-derived growth factor; STAT, signal transducer and activator of transcription; IL-10, interleukin 10; CXCL11, C-X-C motif chemokine 11; CCL18, C-C motif chemokine ligand 18;VEGF, vascular endothelial growth factor.

CAF infiltration in the tumor microenvironment has been correlated with increased invasiveness and poor prognosis of cancer [2,56]. Our data are consistent with a putative contribution of vascular pericytes in the promotion of uveal melanoma invasiveness and progression through pericyte-CAF transition mechanism mediated by PDFG-B (Figure 6).

Uveal melanoma (UM) is the most common primary intraocular tumor in adults, accounting for 5% of all melanomas [57]. In this study, the susceptibility of HRPC to uveal melanoma was first testified by a profound change of cell morphology and phenotype, achieving cancer-activated fibroblast-like phenotype (Figure 1B). This phenotype changing probably represents the sign of a new cellular reprogramming with enhanced proliferative and migratory capability (Figure 2). Moreover, we found that, in response to uveal melanoma, retinal pericytes increased levels of TGF-β1 and vimentin, both considered as CAF markers, and reduced NG2 levels (Figure 1C,D).

### 3.2. In Vitro Interaction with 92.1UM Modulate mRNA Levels of PDGF Isoforms in Both HRPC and Uveal Melanoma

We provide evidence of how uveal melanoma is able to manipulate the pericyte phenotype through the involvement of PDGF-BB. In this in vitro model, we observed greatly increased levels of PDGF-B mRNA, as well as reduced levels of PDGF-A mRNA in HRPC cocultured with 92.1UM. (Figure 1E). These data are consistent with a high production of PDGF-BB dimers by HRPC following the interaction with uveal melanoma. Unlike other PDGF isoforms, the PDGF-BB dimer can bind and activate all PDGFRββ, αβ, and αα dimers, potentiating the autocrine cellular effects of PDGF [58,59]. Moreover, it was hypothesized that different types of receptor dimers are formed depending on the active PDGF isoform, and on which, receptor isoforms are expressed by the target cell [60]. In our model, the pivotal role of PDGF-B in 92.1UM-induced cellular reprogramming of retinal pericytes is confirmed by the unchanged levels of PDGF-C and –D mRNA. We hypothesize that an autocrine mechanism leads to a kind of feed-forward loop between PDGFRβ and PDGF-BB. Imatinib inhibits kinase activities of c-ABL, BCR-ABL, c-KIT, and PDGFRβ activity suppressing the proliferation of cancer cells [61,62]. Chemical blockade of PDGFRβ signaling with imatinib, in our in vitro model, significantly inhibited HRPC capability to reach a CAF-like phenotype counteracting completely the increase of PDGF-B induced by 92.1UM reported in Figure 1B–E. Moreover, we observed a different effect of imatinib in 92.1UM cocultured with HRPC where it was able to increase PDGF-B mRNA levels evoking the mechanisms of tumoral resistance as previously described [63]. Real-time RT-PCR gave us the possibility to discriminate the putative contribution in the PDGF production of both cell lines cocultured. The obtained results indicate the possibility of a mutual interaction between pericytes and uveal melanoma cells, mediated by PDGF-BB, which stimulates a considerable modification of the functions of both cell types.

### 3.3. HRPC Motility Increase Following In Vitro Interaction with 92.1UM

PDGFRs activation mediated by PDGF induces cell proliferation, migration, and differentiation by the involvement of Ras, Erk, MAPK, and PI3K pathways [22,64,65]. In particular, increased cell motility of HRPC induced by 92.1UM can be attributed to chemotaxis that is specifically mediated only by ββ homodimers and αβ heterodimers in smooth muscle cells and fibroblasts [57]. Moreover, the activation of all receptor dimers induced rearrangements of the actin filament system producing circular actin structures, described after ββ- and αβ-receptor dimer stimulation [66]. We did not exclude, in our “in vitro” model, the involvement of dimeric receptor complexes between PDGFR and transforming growth factor β receptor, in order to explain the observed effects on activated retinal pericytes [21]. This mechanism could be relevant in the uveal melanoma-driven transition of retinal pericytes, which express both receptors.

### 3.4. High Levels of PDGF-BB Correlate with Tumor Infiltrating Components and Aggressiveness

Our findings support the possible link between PDGF-BB expression and high content of CAFs in naturally occurring human tumors. In fact, high PDGF-BB expression levels in SC-A431 squamous carcinoma tumor tissue was correlated with a high content of fibroblast-specific protein 1 (FSP1)+ stromal fibroblasts and α-smooth muscle actin (αSMA)+ myofibroblast components. Vice versa, renal cell carcinoma (RCC-CAKI-1) and a neuroblastoma (NB-IMR32) showing undetectable levels of PDGF-BB were characterized by few FSP1+ and αSMA+ fibroblast infiltrating components [10]. The role of PDGF in cancer progression is related to its strong mitogenic and chemoattractant properties for mesenchymal cells, including fibroblasts. Moreover, PDGF can exert its function on tumor cells in a paracrine manner through the involvement of stromal PDGF-sensitive cells, especially fibroblasts in vivo, as indicated by most cancer cells that do not express PDGFR themselves but, nevertheless, display upregulated levels of PDGF [67]. Despite the fact that the secretion of PDGF by tumor cells could not stimulate itself the prosurvival molecules (vascular endothelial growth factor), it leads to the recruitment of fibroblasts that sustain tumor growth and neoangiogenesis [17,68]. Cellular recruitment in the tumor microenvironment represents an important strategy in tumor progression as indicated by the different mechanisms recently described [69]. Gu et al. provide data indicating that exosome-mediated CAF induction is associated with the activation of the TGF-β1/Smad pathway, enhanced proliferative and migratory capacities, and a high expression of the molecular markers FSP, FAP, TSP1, and alpha SMA [70].

### 3.5. 92.1UM-Activated HRPCs are Characterized by a Tumor-Supporting Pattern of Cytokine and Chemokine mRNA Levels

Functional changes of cocultured HRPC with 92.1UM were documented by evaluating the mRNA levels of tumor-promoting cytokines and chemokines. Interestingly, what we observed was an “as defined” immunomodulatory cytokine profiling as indicated by high levels of TGF-β1 (studied at protein levels, in Figure 1C, and at mRNA levels, in Figure 3) and mostly by high levels of IL10 (Figure 3) [17]. An immunomodulatory balance of cytokines was associated with an immunoescape capability of tumors in vivo [17]. Increased levels of chemokines, CXC11 and CCL18, and VEGFA mRNA levels in cHRPC further confirmed the tumor-supporting role of HRPC towards 92.1UM probably through different molecular strategies, i.e., (i) promoting monocyte chemotaxis [50,54] and (ii) stimulating angiogenesis. These preliminary data indicate that the activated HRPC could contribute to tumor progression and spread.

### 3.6. STAT3 Nuclear Translocation Mediate HRPC-CAF Transition

Here, we propose, for the first time, that retinal pericyte activation into CAFs is mediated by STAT3 phosphorylation and nuclear translocation following uveal melanoma interaction (Figure 6) as indicated by the increased number of positive nuclei for phospho-STAT3 in HRPC cocultured with 92.1UM (Figure 4D,E). Our data support the idea that JAK/STAT3 signaling plays a pivotal role in transcriptional reprogramming associated with pericyte recruitment and activation after tumor interaction. Many reports in the literature describe the role of JAK/STAT3 signaling in gene expression reprogramming that bears epithelial–mesenchymal transition regulating tumor metastasis, the transition of cancer stem cells and chemoresistance [71]. New efforts will be necessary to elucidate cell-interaction mechanisms between retinal pericytes and uveal melanoma that, in turn, induce PDGF-B upregulation. However, we provide evidence about a functional involvement of uveal melanoma in terms of increased invasiveness following interaction with conditioned cHRPC. As shown in Figure 5, exposure of HRPC to 92.1UM elicits their capability to induce the invasiveness of uveal melanoma in a typical invasion test conducted using Boyden chambers. We correlate these observations to the increased levels of active metalloproteinase MMP9 indicating a reciprocal cellular interaction between retinal pericytes and uveal melanoma that induces effective cellular responses also in the tumor. “In vitro” models of cell cultures based on coculture provided evidence of being very effective for studying the involvement of diffusible factors in biological and pathological processes.

### 3.7. Limitations and Weaknesses of the Study

In vitro differentiation of pericytes is affected by the experimental condition as they can spontaneously dedifferentiate in different mesenchymal cell types (e.g., adipocytes, chondrocytes, osteoblasts, fibroblasts, and VSMCs) [72,73]. In a standard culture media, pericytes show a low proliferative rate and undergo early senescence. Here, we cannot exclude any potential impact of the coculture medium (PM + 10%FBS RPMI-1640, 50:50 ratio, for 6 days) used in order to ensure the better culture condition for both cell lines. Moreover, we do not address the role of physical contact with endothelial cells in the activation of HRPC induced by 92.1UM as it modulates pericyte phenotype and the response to different growth factors present in their environment. Pericytes show heterogeneous characteristics in term of density, morphology, and function in different vascular beds. The contribution of vascular pericyte heterogeneity to cancer progression and therapy response needs to be further investigated. Certainly, our experimental in vitro findings need to be validated through future in vivo studies in order to address the role of other critical elements that may affect in vitro responses, such as extracellular matrix composition, cell density, and cell passage.

## 4. Materials and Methods

### 4.1. Reagents

Rabbit polyclonal antibody against phospho-PDGFRβ (catalog n. sc-21902) and goat polyclonal antibody against PDGFRβ (catalog n. sc-1627) were purchased from Santa Cruz Biotechnology, Inc. (Santa Cruz, CA, USA). Rabbit monoclonal antibody (EP1255Y) against MMP9, rabbit polyclonal antibody against NG2 (catalog num. ab129051), rabbit monoclonal antibody (E184) against α smooth muscle Actin (α-SMA, catalog n. ab32575), rabbit polyclonal antibody against transforming growth factor beta-1 proprotein (TGF-β1, catalog n. ab66043), and rabbit monoclonal (EPR3776) against vimentin (catalog n. ab92547) were purchased from Abcam (Cambridge, UK). Rabbit polyclonal antibody against fibroblast-specific protein 1 (S100A4) (catalog n. ABF32) was purchased from Millipore (Darmstadt, Germany). Mouse monoclonal antibody (BA3R) against β–actin (catalog n. MA5-15739) was purchased from Invitrogen, Thermo Fisher Scientific (Waltham, MA, USA). Secondary goat antirabbit IRDye 680 conjugated antibody (catalog n. 926-32221) and secondary goat antimouse IRDye 800 conjugated antibody (926-32210) were purchased from LI-COR (Lincoln, NE, USA). Imatinib (Glivec) was obtained from Novartis (Basel, Switzerland). Media, antibiotics, and other reagents for cell culture were obtained from Invitrogen, Thermo Fisher Scientific.

### 4.2. Cell Cultures

Primary human retinal pericytes (HRPC) were purchased from Innoprot (Elexalde Derio, Spain). HRPC were fed with PM culture medium, supplemented with 2% fetal bovine serum (FBS), 1% PGS (Pericyte Growth supplement), 100 U/mL penicillin, and 100 μg/mL streptomycin. Culture medium and supplements were purchased from Innoprot. Cells were plated in T25 culture flasks (Costar; Corning, New York, NY, USA), precoated with 0.01 mg/mL poly-L-lysine (Innoprot) for 1 h at 37 °C. Cells were incubated at 37 °C with 5% CO_2_ until reaching about 70% confluence. Further, 92.1 human uveal melanoma cell line was purchased from the Cell Factory-IST (Genoa, Italy) and grown in monolayer cultures in RPMI-1640 medium supplemented with 10% fetal bovine serum (FBS), 100 IU/mL penicillin, 100 µg/mL streptomycin, and 2 mmol/L of L-glutamine in a humidified atmosphere (5% CO_2_) at 37 °C. All experiments were carried out using cells at passage 3–4.

### 4.3. HRPC Conditioning by 92.1UM in an “In Vitro” Model of Coculture

HRPC were seeded in poly-L-lysine precoated 6 or 12 well plates at a density of 4.3 × 10^3^ cell/cm^2^ and were grown in complete PM medium overnight at 37 °C with 5% CO_2_. Briefly, 92.1UM were seeded into 24 or 12 mm format Transwell inserts (pore sizes 1 μm, Corning, Inc.) at a density of 5 × 10^3^ cell/cm^2^ and were grown in 10% FBS RPMI-1640 medium overnight at 37 °C with 5% CO_2_. The choice of multiwell and insert formats depended on the number of cells required for downstream applications. Then, cocultures were created when HRPC wells that received 92.1UM inserts for 6 days in order to allow pericyte conditioning (conditioned HRPC, cHRPC) by diffusible factors mutually produced. Coculture medium consisted of both complete PM and 10% FBS RPMI-1640 (50:50 ratio). In parallel, some cocultures were performed in the presence of 5 μM of imatinib (conditioned HRPC plus imatinib, c/IMAHRPC). Pericytes grown without 92.1UM inserts for 6 days in coculture medium were considered as control cells HRPC. Coculture media, with or without imatinib, were replaced every 72 h. After 6 days of coculture, cells were washed in phosphate-buffered saline (PBS) 1x and were recovered by trypsinization for further analyses.

### 4.4. Wound Healing Assay

HRPC migration was measured using a standard wound healing assay. After the conditioning period, the 92.1UM inserts were removed from 12 wells and monolayers of confluent HRPC, cHRPC or c/IMAHRPC were scratched with a P200 pipette tip. Then, cells were washed with PBS 1x and incubated with fresh serum-free coculture medium (serum-free PM and RPMI-1640, 50:50 ratio). Wound closure was monitored by photographs at 40× using a phase-contrast microscope. Wound closure was evaluated in each culture condition and at each time point (0, 24, and 48 h) by taking images from randomly selected fields. HRPC migration was quantified by measuring the distance travelled over time by both cell fronts into the wound area using ImageJ software program.

### 4.5. Proliferation Assay

Cell proliferation was measured using crystal violet staining of HRPC after conditioning in 12-well plate with inserts containing 92.1UM without or with 5 µM of imatinib. At the end of the conditioning period, inserts were removed and each well was washed with phosphate-buffered saline (PBS). Following this, cells were fixed and stained with 0.5% crystal violet solution in 20% methanol for 10 min. Subsequently, the plate was washed with water and left to dry. Each assay was carried out in triplicate. Trypan blue staining was evaluated by measuring the absorbance at 570 nm with the microplate reader (Synergy 2-BioTek).

### 4.6. Extraction of Total RNA and Real-Time Reverse Transcriptase-Polymerase Chain Reaction (RT-PCR)

Total RNA was extracted using TRIzol reagent (Invitrogen Life Technologies), according to the manufacturer’s instructions and redissolved in 30 μL RNase-free water. RNA concentration and purity were estimated by optical density measured at 260 and 280 nm. First-strand cDNA was synthesized by reverse transcription (RT) of 1 µg RNA in a 20 μL reaction volume with 200 U of SuperScript III, 50 ng random hexamers, 1.25 mM dNTP, 10 mM dithiothreitol, 50 mM Tris-HCl, 75 mM KCl, and 3 mM MgCl_2_ (Thermo Fisher Scientific, Carlsbad, CA, USA), pH 8.3. The reaction, carried out at 50 °C for 50 min, was stopped at 85 °C for 5 min. Aliquots of 5 ng cDNA were amplified in parallel reactions using Quant Studio 3 Applied Biosystems, Thermo Fisher Scientific (Waltham, MA, USA). Each PCR reaction (10 μL final volume) contained 0.8 μM of forward and reverse specific primers, 1X iTaq™ Universal SYBR^®^ Green Supermix (Bio-Rad Laboratories, Milan, Italy) and 1 µL of cDNA. Forty amplification cycles were carried out for each sample. Results were analyzed with the 2^−ΔΔ^Ct method. The results were normalized by product of 18S ribosomal RNA (rRNA) gene expression. Primers were purchased from Eurofins Genomics (Milan, Italy). Primer sequences, product size, annealing temperature, and gene bank accession number are listed in Table 1. The specificity of the PCR reaction was assessed by the denaturation temperature of the amplification products [49].

### 4.7. High-Content Screening (HCS) and Image Analysis

High-content screening was used to evaluate the amount of phospho-PDGFRβ, and total PDGFRβ HRPC were seeded in 12-well plate and conditioned as described in Section 2.3. At the end of the conditioning period using 92.1UM, pericytes were washed three times with PBS and fixed with 4% paraformaldehyde for 30 min at 4 °C. Permeabilization and blocking were carried out by incubating cells for 30 min in a 5% solution of normal goat serum and 0.2% Triton X in PBS. Then, cells were incubated overnight at 4 °C with primary antibodies in a PBS/Triton 0.1% solution. All primary antibodies were tested at a concentration of 1:150. After incubation, cells were washed tree times with 0.2% Tween 20 in PBS solution and were then incubated with the appropriate secondary antibody in PBS (1:200 concentration) for 1 h at RT. After removing the secondary antibody, samples were washed three times in PBS and the nuclei were stained with NucBlue solution for 15 min at RT following the manufacturer’s instructions (Thermo Fisher Scientific). Cells were imaged using the PerkinElmer Operetta High-Content Imaging System (# HH12000000). Plates were read under confocal conditions using the 20× long WD objective. Different fluorescence type channels were used to acquire images of NucBlue (Ex: UV light, Em: 460 nm) for nuclei staining, shown in blue; TRITC (Ex: 557 nm, Em: 576 nm) for phospho-STAT3 staining, shown in red; Alexa Fluor 488 (Ex: 496 nm and Em: 519 nm) for phospho-PDGFRβ, shown in green; and Alexa Fluor 546 (Ex: 556 nm and Em: 573 nm) for total PDGFRβ, shown in red. Enough fields were imaged to capture at least 200 cells per well. All images were analyzed using Harmony high-content imaging and analysis software (PerkinElmer). Initial segmentation of cells was carried out in the DAPI channel by identifying the blue-stained nuclei with an area > 30 µm and cytoplasm as well as membrane were defined in segmented cells on the basis of the different stainings used. For PDGFRβ protein level analysis, we defined intensity (mean per well) of Alexa Fluor 488-related green fluorescence (corresponding to phospho-PDGFRβ staining) and Alexa Fluor 546-related red fluorescence (corresponding to total PDGFRβ staining) using the *Calculate Intensity Properties* function in cytoplasm and membrane. For the analysis of phospho-STAT3 nuclear translocation, after nuclei identification, we evaluated the intensity of the TRITC channel (corresponding to phospho-STAT3 staining) in the nuclei population of the control HRPC using the *Select Population* function. Subsequently, we set a threshold value of fluorescence as TRITC channel intensity up to 95° percentile of the nuclei population in control HRPC. This threshold value was used to calculate the percentage of translocated nuclei in the other experimental conditions, considering all nuclei positive (i.e., translocated) with a mean signal intensity for TRITC greater than the threshold value. Finally, the percentage of positive nuclei for phospho-STAT3 was calculated using the *Calculate Properties* function with the formula: A/B × 100, where A is the number of positive nuclei and B is the number of analyzed objects (cells). Final output values from the analysis were expressed as mean per well.

### 4.8. Western Blot Analysis

Proteins of whole cell lysates were extracted with RIPA buffer (with protease and phosphatase inhibitor cocktail) (Sigma-Aldrich, St. Louis, MO, USA). Extracted proteins (30 µg) were loaded on 4–20% precast polyacrylamide gel (Mini-PROTEAN^®^ TGX™ Precast Protein Gels, Bio-Rad Laboratories, Milan, Italy) and, afterwards, transferred to nitrocellulose membranes. Immunoblot was preceded by 30 min incubation with Odyssey Blocking Buffer (LI-COR Biosciences, Lincoln, NE, USA), and subsequently, the membranes were incubated at 4 °C overnight with an antibody against TGF-β1 (1:200), IL-10 (1:800), vimentin (1:2000), MMP9 (1:1000), NG2 (1:500), and phosphor-PDGFRβ (1:250). β-actin served as the loading control. Immunoblot was detected through Odyssey Imaging System (LI-COR Biosciences, Lincoln, NE, USA). Densitometry analyses of blots were performed using the ImageJ software Version 1.43, (Broken Symmetry Software, Bethesda, MD, USA).

### 4.9. Invasion Assay of 92.1UM Cells

Cell invasion assay was performed using Boyden chambers consisting of Matrigel-coated Transwell Cell Culture (8 μm pore size) following the manufacturer’s instructions (Cat. Num 354481, BD biosciences, Bedford, MA, USA). This membrane represents the in vivo extracellular matrix. Briefly, pericytes were plated into 6-well plates at a density of 4.3 × 10^3^ cell/cm^2^ in 2% FBS and were conditioned as indicated in Section 2.3, the conditioning period was stopped after 6 days by replacing coculture medium of each well with serum-free coculture medium. Then, HRPC, cHRPC, and c/IMAHRPC wells received the Boyden chamber with fresh seeded 92.1UM at a density of 4 × 10^5^ cell/well previously starved for 1 h. Chemoattractant (RPMI 10% FBS) was added to the lower chamber and used as a positive control, whereas PBS was used as a negative control. We allowed cells to migrate into the lower chamber for 24 h, by actively degrading chambers Matrigel, mimicking invasion of an organ. Noninvaded cells were removed by a cotton swab and chambers were washed with PBS. Cells that migrated throughout the membrane were fixed in methanol for 10 min, rinsed in water, and stained using crystal violet. The basolateral side of the Boyden chamber with stained cells was observed under an inverted bright field microscope at 20× magnification. Cell number of randomly selected fields was evaluated by direct counting. Invasion was also quantified through the solubilization of retained crystal violet from migrated 92.1UM by SDS 1X. Then, 100 μL of solubilized crystal violet solution was evaluated by measuring the absorbance at 570 nm with the microplate reader (Synergy 2-BioTek) and was indicated as invasion index.

### 4.10. Statistical Analysis

All results are reported as mean ± SEM. The results were analyzed using one-way ANOVA followed by Tukey–Kramer multiple comparisons test; differences between groups were considered significant for *p*-value < 0.05.

## 5. Conclusions

Here, we described a mechanism of cellular crosstalk between uveal melanoma and retinal pericytes based on the production of diffusible factor PDGF-B, leading to pericyte transition into cancer-activated fibroblasts (CAF) in an in vitro model of coculture. A signaling pathway, involving STAT3 translocation could mediate pericyte activation, as indicated by the upregulation of mRNA levels of IL-10, TGF-β1, CCL10, CXCL11, and VEGF. Activated pericytes triggered the increase of uveal melanoma invasiveness through the upregulation of the active MMP9 isoform. Imatinib, by inhibiting PDGFRβ activation and, consequently, CAF transition, could be able to counteract uveal melanoma invasiveness.

## Figures and Tables

**Table 1:** Primer sequences used for quantitative PCR.

Gene	Sequence (5’-3’)	Amplicon (bp)	Accession Number
VEGFA	Fw: ATCTTCAAGCCATCCTGTGTGC	121	NM_001025366.3
	Rv: GAGGTTTGATCCGCATAATCTG		
*CCL18*	Fw: CTCCTTGTCCTCGTCTGCAC	248	NM_002988.4
	Rv: TCAGGCATTCAGCTTCAGGT		
CXCL11	Fw: GCCTTGGCTGTGATATTGTG	235	NM_001302123.2
	Rv: TGATTATAAGCCTTGCTTGCTTCG		
IFN- γ	Fw: AGATGACTTCGAAAAGCTGACT	95	NM_000619.3
	Rv: ACAGTTCAGCCATCACTTGG		
IL10	Fw: GACTTTAAGGGTTACCTGGGTTG	112	NM_000572.3
	Rv: TCACATGCGCCTTGATGTCTG		
IL-1 β	Fw: AGCTACGAATCTCCGACCAC	186	NM_000576.3
	Rv: CGTTATCCCATGTGTCGAAGAA		
TGF- β1	Fw: CGTCTGCTGAGGCTCAAGT	74	NM_000660.7
	Rv: CGCCAGGAATTGTTGCTGTA		
TNF-α	Fw: AGCCCATGTTGTAGCAAA CC	134	NM_000594.4
	Rv: TGAGGTACAGGCCCTCTGAT		
18S rRNA	Fw: TAAGTCCCTGCCCTTTGTACACA	69	NR_146119
	Rv: GATCCGAGGGCCTCACTAAAC		
